# Annotations

**Published:** 1907-01-05

**Authors:** 


### ANNOTATIONS.

### Pharmacopœial Revision.

THE issue of a report to the Pharmacopœia Committee of the General Medical Council by the Committee of Reference in Pharmacy, is a practical illustration both of the vigilant criticism which attends the life of a pharmacopœia and of the keen eye which is kept on such criticism by those responsible for the structure of the official volume. It is impossible to turn over the pages of this report without being impressed with the amount of work which is constantly in operation with a view to maintain and increase the value and accuracy of the pharmacopœial drugs and their preparations. It is now eight years since the last edition of the Pharmacopœia was issued, and doubtless strength and material are being accumulated for the composition of a new issue. For the most part, it is to be feared that the critics of the pharmacopœial work, as is the habit of their kind, are attracted more by faults than by virtues. Hence we welcome an opportunity of expressing our appreciation of the value of the sustained attention and constant preparation which are steadily supplied by the responsible committee. Especially in the present report is to be seen the value of the co-operation of the expert pharmacists whose assistance is essential on so many points. The melting point of a fat, an exact estimate of an ash percentage, a slight difference in the degree of solubility—these and other details may perhaps seem unimportant matters to the busy practitioner; but insistence on such details is an essential part of the machinery by means of which the Pharmacopœia is kept as the uniform standard and guide for the composition of medicines in the British Isles. Relaxation here would mean the risk of carelessness in other directions, and, so far from disregarding this critical work, we are refreshed by its fineness and vigour. It represents a portion of the debt which medicine owes to the art of pharmacy.

### Dr. F. Roberts on Baths and Bathing.

IN his interesting address to the Balneological and Climatological Society, Dr. Frederick Roberts made some incidental references to the daily bath, which, at least among some classes, is part of the daily toilet of the Englishman. Perhaps the dominant note in his discussion of this subject is to be found in the phrase "the personal factor must not be ignored." That is the qualification which limits so many of the would-be dogmatic general rules of medical practice. There will, of course, be no difficulty in securing medical assent to the proposition that boys and girls should be educated to look upon a daily bath as a duty they owe to themselves and to society. Nor will there be hesitation in endorsing the remark that infants and young children require tender treatment in the matter of baths, and that this gentleness implies more or less warmth. But differences of opinion arise when the question of the temperature of the daily bath for the middle-aged or elderly adult is discussed, and we are particularly glad to find Dr. Roberts arguing against the virtues of the cold bath at these periods of life. For vigorous and active youth the cold tub is a valuable and tonic discipline. Even some adults who have reached or passed middle life practise it with impunity, or even with advantage. But for most men of sedentary habits the cold tub is not suitable. It has a heroic and Spartan aspect, and, being more or less disagreeable, is often therefore argued to be virtuous. Popularly hot water is supposed to "open pores" or to "relax fibres," to use the pseudo-scientific jargon of the day, and so to provoke to "catching cold" and other evils. The question in any individual case must be settled by actual experience, but there can be no doubt that many a middle-aged man would enjoy both better health and better temper if he took a warm instead of a cold bath. Personal comfort and domestic peace often have a secret and unsuspected enemy in the chilling influence of the morning tub.

### Mountain Sickness.

FOR some time there was a good deal of mystery in regard to this morbid state and the causes underlying it, and there seems little doubt that the term was applied to varying complex groups of symptoms which were largely due to other conditions than the actual elevation above the sea-level and the attending atmospheric changes. Zuntz and his pupils have recently carried out extensive observations in the laboratory and above the snow line, with a view to determine the physiological changes in the organism resulting from diminished atmospheric pressure and lessened oxygen tension, and Dr. Longstaff has lately reviewed the whole subject in an interesting monograph. After quoting the varied experiences of mountaineers as far back as A.D. 1519, he shows conclusively that the fatigue consequent on the continual and arduous work of mountain climbing forms a most important factor in the causation of mountain sickness, and probably largely accounts for the fact that the condition frequently occurs in a severe form at the comparatively low altitudes of 9,000 to 15,000 feet, altitudes which are attained by inexperienced and untrained mountaineers, and at which the atmospheric pressure is only reduced to about two-thirds, whereas the more highly trained have reached heights of over 20,000 feet, or less than half an atmosphere, without experiencing any greater inconvenience than some breathlessness on exertion and weakening of muscular power. Muscular weakness and acceleration of cardiac and respiratory rhythm, combined with anorexia and headache form the group of symptoms to which the term mountain sickness is applicable, and these appear to be caused by a deficient supply of oxygen consequent on the diminished atmospheric pressure. Experiments in the pneumatic chamber confirm the view that the condition arises from the diminished oxygen tension, and not from the low pressure of the atmosphere itself. It is, in fact, a condition of oxygen hunger. The experience of mountaineers shows that a prolonged stay at high altitudes does not lead to acclimatisation to the altered conditions, but, on the contrary, tends to a continued diminution in muscular power, and a general deterioration in health.

